# Supplementary material for: Xenon plasma focused ion beam lamella fabrication on high-pressure frozen specimens for structural cell biology
Source: Nat Commun. 2025 Mar 7;16:2286. doi: 10.1038/s41467-025-57493-3 (PMC11889171; doi:10.1038/s41467-025-57493-3)
Supplement: Supplementary file 1 — Supplementary Information [file 41467_2025_57493_MOESM1_ESM.pdf]

# Xenon plasma focused ion beam lamella fabrication on high-pressure frozen specimens for structural cell biology

Casper Berger[1]*, Helena Watson[1,2]*, James Naismith[1,3], Maud Dumoux[1], Michael Grange[1,4,‡]

[1]Structural Biology, The Rosalind Franklin Institute, Harwell Science & Innovation Campus, Didcot OX11 0QS, United Kingdom.
[2]School of Molecular Biosciences, College of Medical Veterinary and Life Sciences, University of Glasgow, Glasgow, UK
[3]Mathematical, Physical and Life Sciences Division, University of Oxford, Oxford, UK
[4]Division of Structural Biology, Wellcome Centre for Human Genetics, University of Oxford, OX3 7BN Oxford, United Kingdom.

*These authors contributed equally
‡To whom correspondence should be addressed: michael.grange@rfi.ac.uk

## Supplementary Information

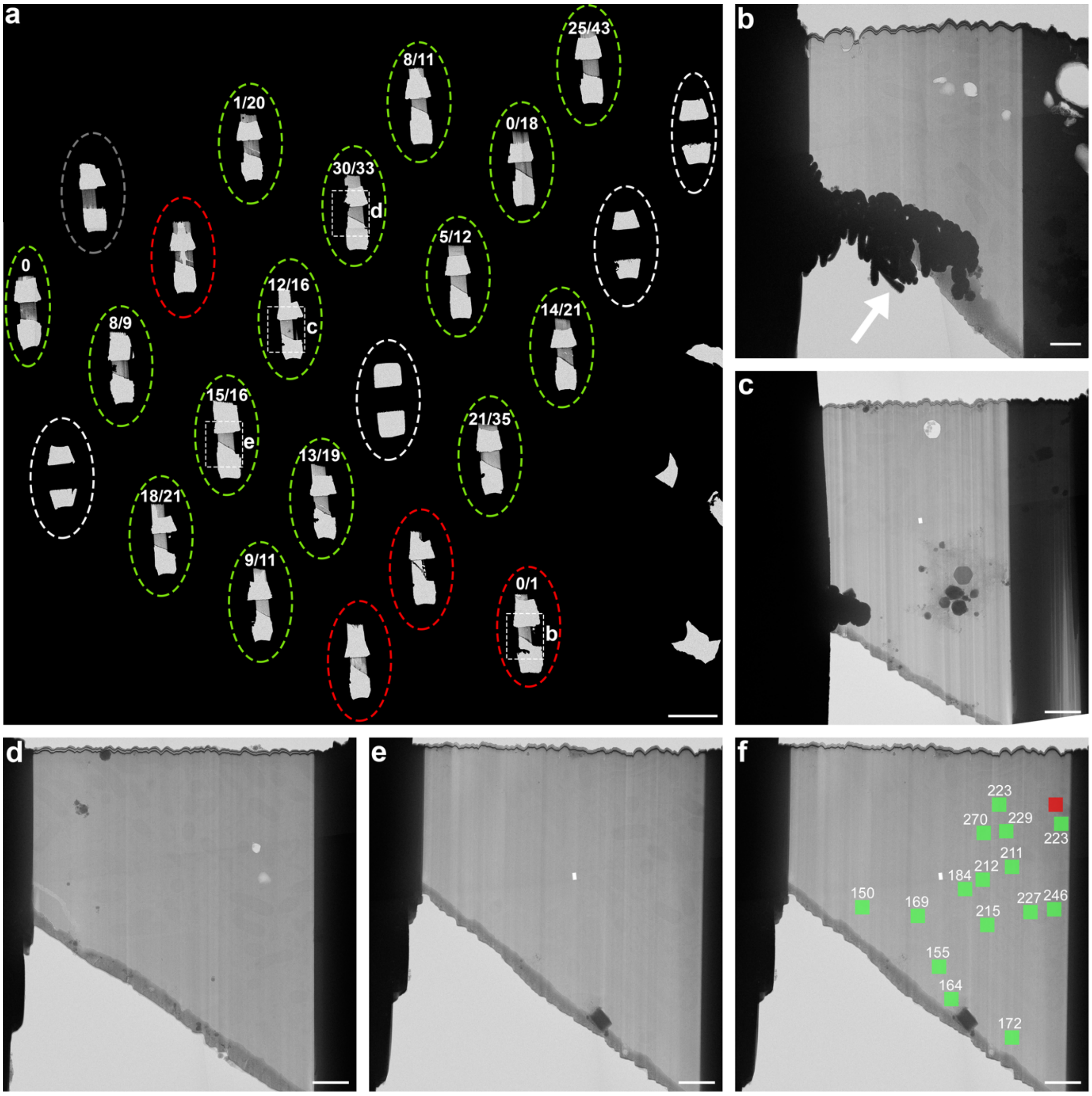


**Supplementary Figure 1. *E. coli* TEM grid and lamella overviews. a** Stitched TEM atlas image collected at 0° stage tilt of a grid containing the 24 lamellae sites indicated in Fig. 2a. Sites that were not further used for automated lamella fabrication are indicated with white dashed ellipses, lamellae considered unsuitable for tilt-series acquisition due to lamella breakage or insufficient removal of material below the lamellae are indicated with red dashed ellipses and lamellae suitable for tilt-series acquisition (clear biological contrast in low-dose TEM overviews on a stable lamella, without objects blocking the beam at high tilts) indicated with green dashed ellipses. One lamella manually polished for test purposes is indicated with a grey dashed ellipse. The number of acquired tilt-series on each lamella, and the number used for STA is indicated in white above each site, and the white letters on the right site of some sites indicate that low-dose TEM overviews are shown in the respective panel b-e. Scalebar: 50 µm**. b-e** Example low-dose TEM overview of lamellae unsuitable (b) and suitable (b-d) for tilt-series acquisition collected at 20° stage tilt. Lamella in shown in b was excluded because not all the material was removed below the lamella (white arrow). Scalebars: 2 µm. **f** The same lamella overview as shown in panel e, with squares indicating the approximate position were tilt-series were acquired that were used for subtomogram averaging (green) or excluded from further processing (red). The measured tomogram thickness (nm) for each site is shown in white. Scalebars: 2 µm. Source data are provided as a Source Data File.

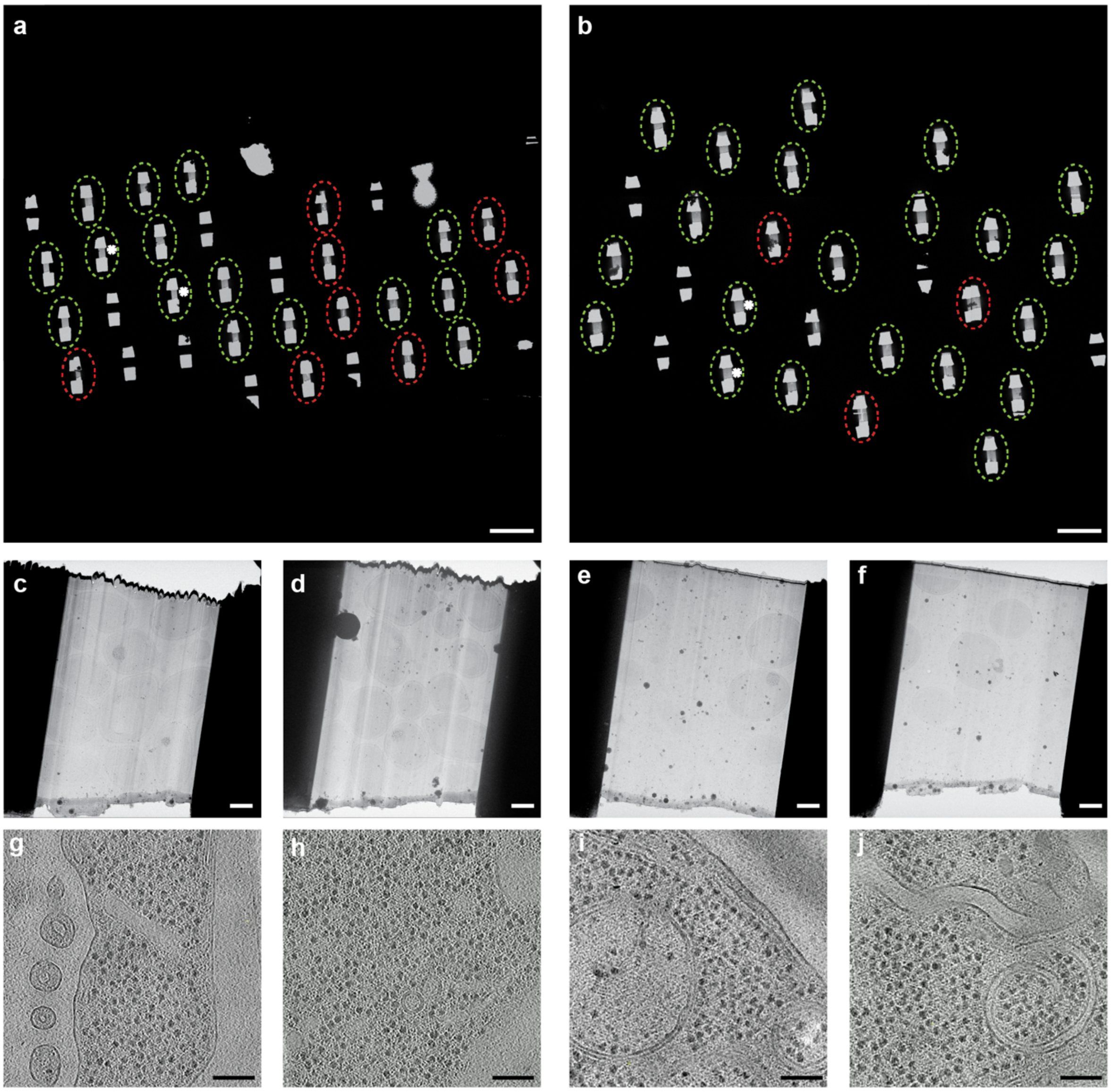


**Supplementary Figure 2. *S. cerevisiae* TEM grid and lamella overviews**. **a, b** Stitched TEM atlas images collected at 0° stage tilt of the *S. cerevisiae* grids indicated in Fig. 2a. Lamellae considered unsuitable for tilt-series acquisition due to lamella breakage or insufficient removal of material below the lamellae are indicated with red dashed ellipses and lamellae suitable for tilt-series acquisition indicated with green dashed ellipses. Scale bars: 100 µm. **c-f** Example low-dose TEM overview of lamellae suitable for tilt-series acquisition collected at 20° stage tilt. Lamellae of (c, d) and (e, f) are from the grids shown in (a) and (b) respectively. Scale bars: 2 µm**. g-j** Examples of reconstructed slices of *S. cerevisiae* tomograms. Tomograms (g, h) and (i, j) are from the grids shown in (a) and (b) respectively. Scale bars: 100 nm.

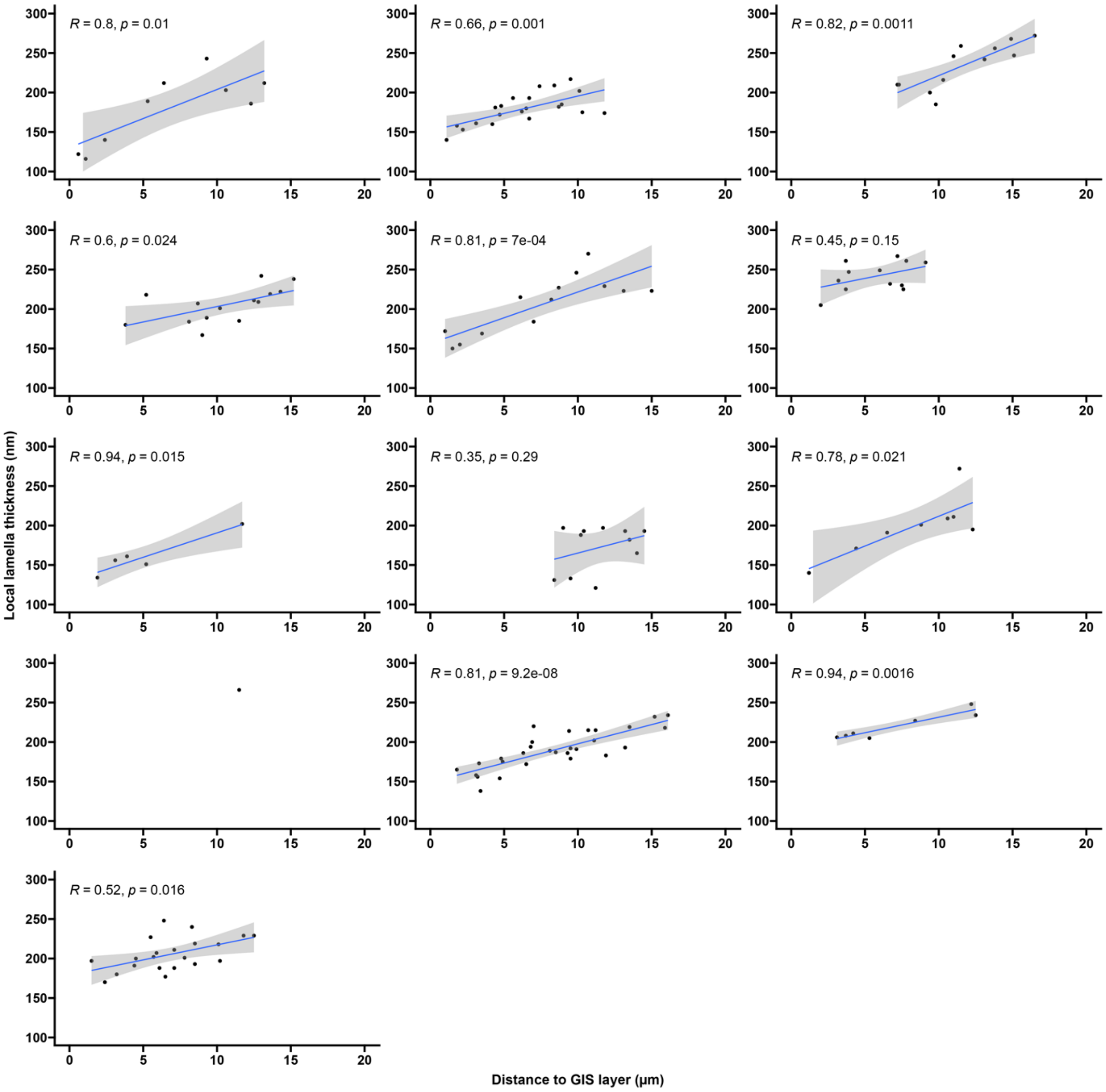


**Supplementary Figure 3. Plots for local lamella thickness in relation to distance from the front of the lamella.** Scatter plots for local lamella thickness measured in the tomograms used for damage analysis compared to the distance to the front of the lamellae. A linear trend line (blue) is plotted for each lamella with the 0.95 confidence interval (grey) and the Pearson correlation (r) and p-value (p) are shown for each lamella. Source data are provided as a Source Data File.

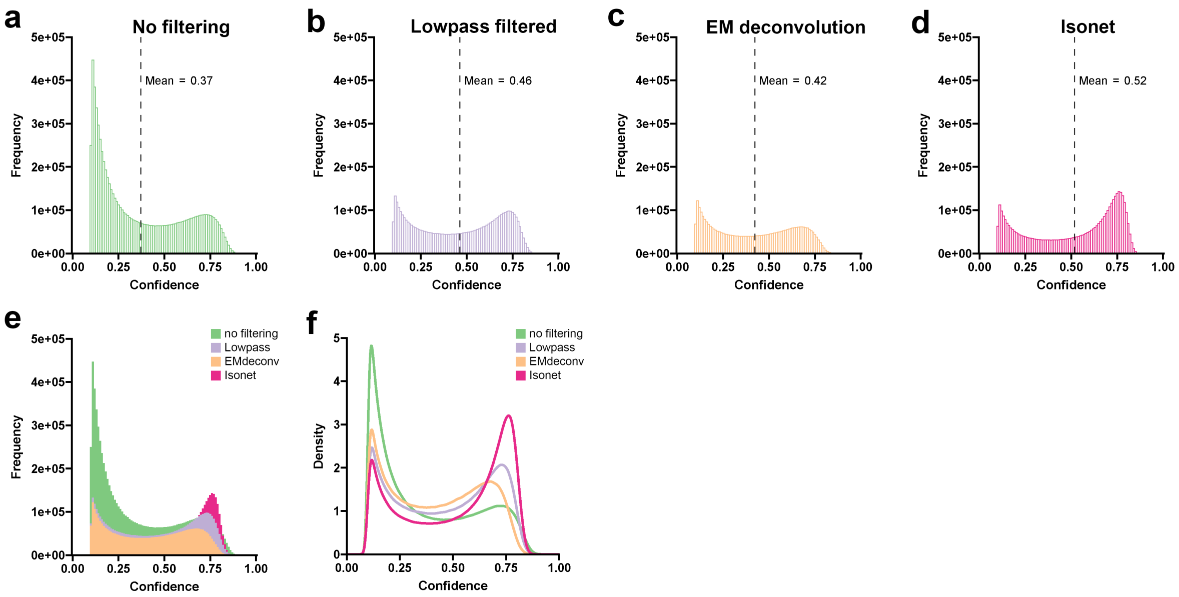


**Supplementary Figure 4. Effects of filtering on effectiveness of automated ribosome particle picking.** Distribution of crYOLO-assigned confidence scores for machine-learning based picking of ribosomes with crYOLO[40] on filtered or unfiltered tomograms. **a-d** Histograms (bin width 0.01) showing the frequency of the confidence scores during prediction for ribosome positions identified in 2D slices of tomograms that were: SART reconstructed tomograms without additional filtering (a), lowpass filtered (b), EM deconvolution filtered (c) or filtered using machine-learning based missing-wedge compensation as implemented in Isonet[38]. Values for the mean are shown in the plots and indicated with vertical black dashed lines. A total of 8.01 x $10^6$ 2D ribosome positions were identified on unfiltered tomograms, 4.70 x $10^6$ for lowpass filtered tomograms, 3.65 x $10^6$ for EM deconvolved tomograms and 4.37 x $10^6$ for Isonet filtered tomograms. **e** Overlay of the histograms shown in panel a-d. **f** Density plots for the confidence scores of the same four conditions. Source data are provided as a Source Data File.

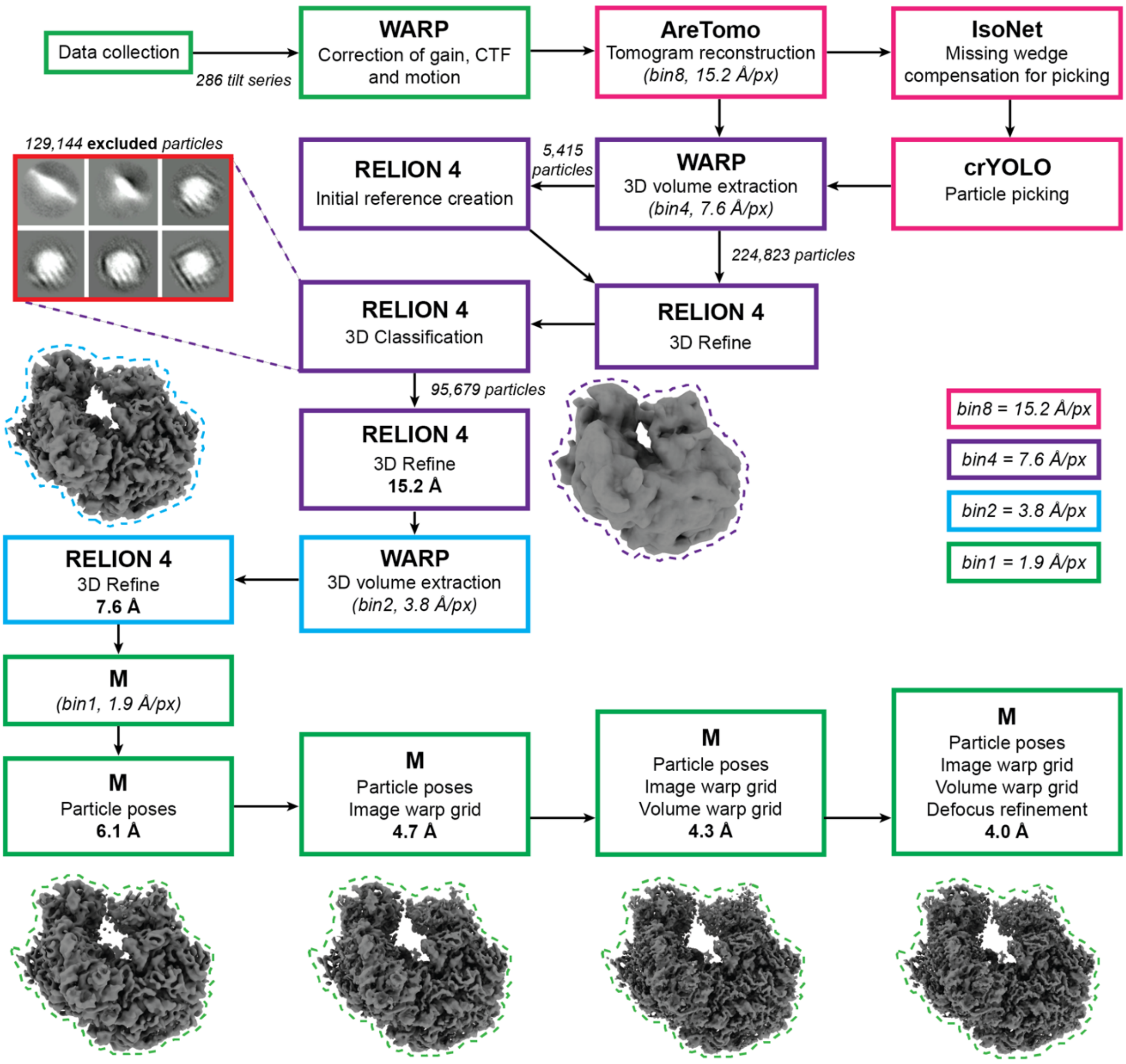


**Supplementary Figure 5. Overview of STA processing pipeline.** Coloured frames indicate the binning factor used during each processing step and the obtained resolutions of the depicted refined density maps are indicated in bold.

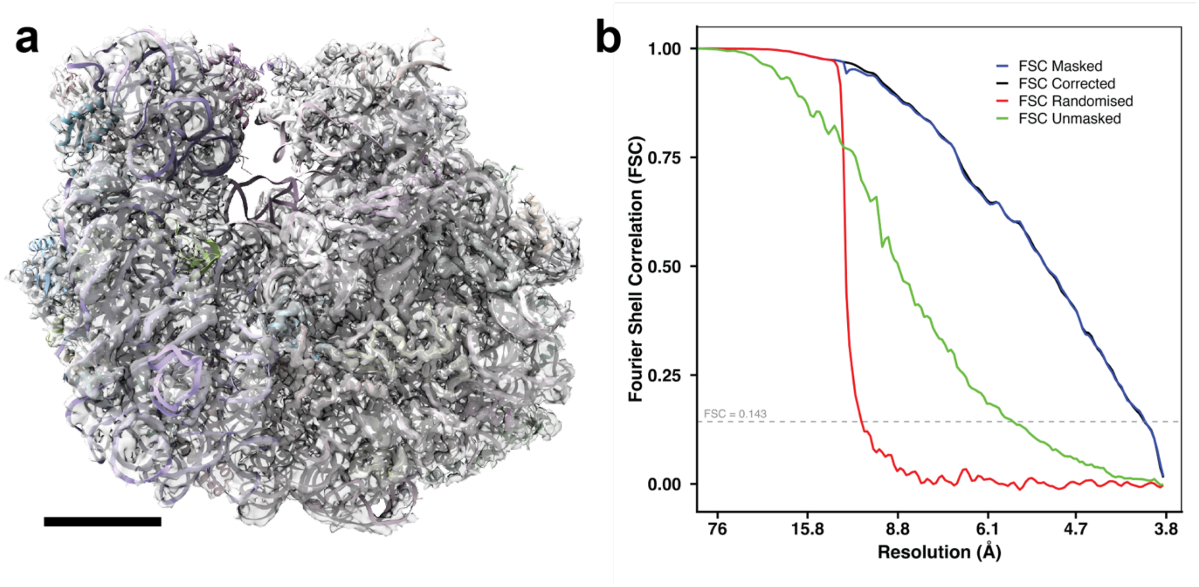


**Supplementary Figure 6.** ***In situ*** **STA structure of the** ***E. coli*** **70S ribosome.** **a** Consensus STA density map of the 70S ribosome with fitted cryo-EM atomic model (PDB: 6ORE)[45]. Scale bar: 5 nm **b** Fourier Shell Correlation gives masked global resolution of 4.0 Å. Source data are provided as a Source Data File.

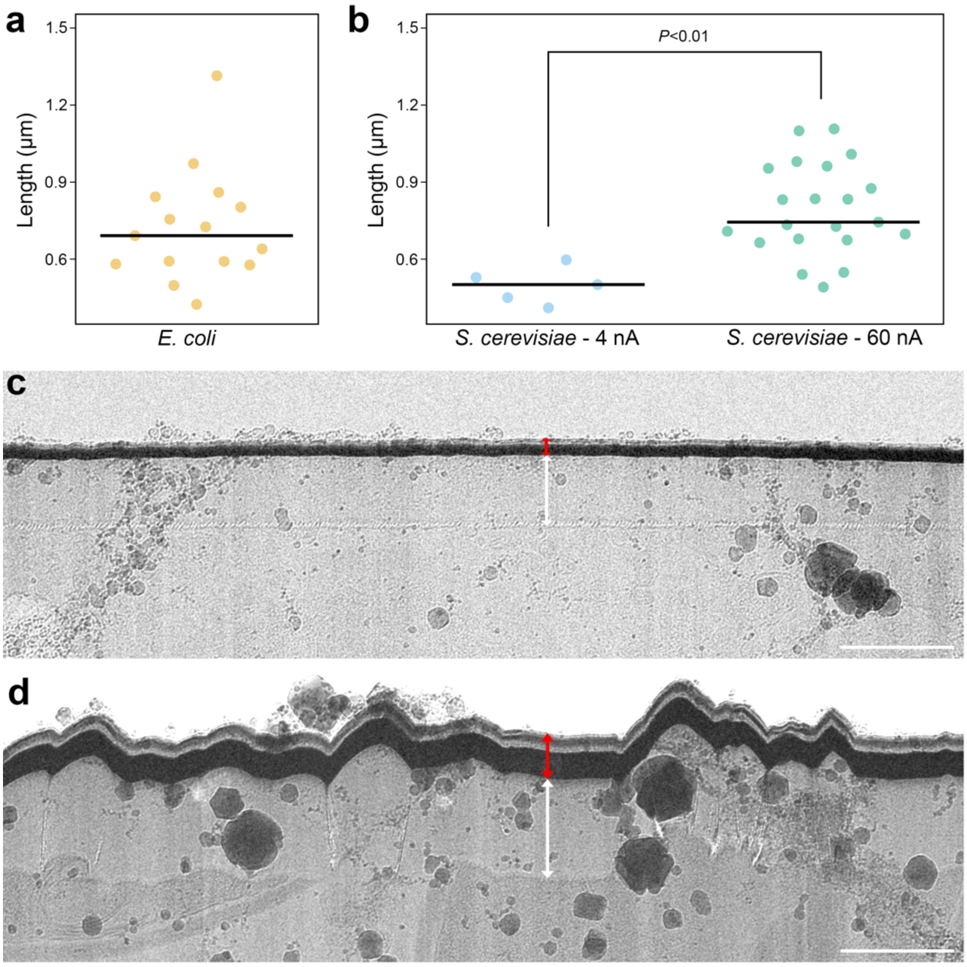


**Supplementary Figure 7. The effect of milling current on the length of the backside amorphous layer**. **a** Scatter plot showing the distribution of the length of the amorphous layer (white arrows in panel c and d) on the backside of lamellae prepared on *E. coli* using 60 nA for the first trench milling step (mean SD; 0.72± 0.22 µm, n=15). Median (0.69 µm) is indicated with a horizontal black line. **b** Scatter plot showing the distribution of the length amorphous layer on the backside of lamellae prepared on *S. cerevisiae* using 4 nA (mean SD; 0.5± 0.07 µm, n=5) or 60 nA (mean SD; 0.79± 0.18 µm, n=21) for the first trench milling step, where 4 nA resulted in a significantly lower length of the amorphous area (two-sided t-test: $p$ = 0.0012). Median values (0.5 and 0.74 µm) are indicated with horizontal black lines. **c, d** Example TEM overviews of lamella prepared with 4 nA (c) or 60 nA (d) for the first trench milling step. Scalebar: 1 µm. Source data are provided as a Source Data File.

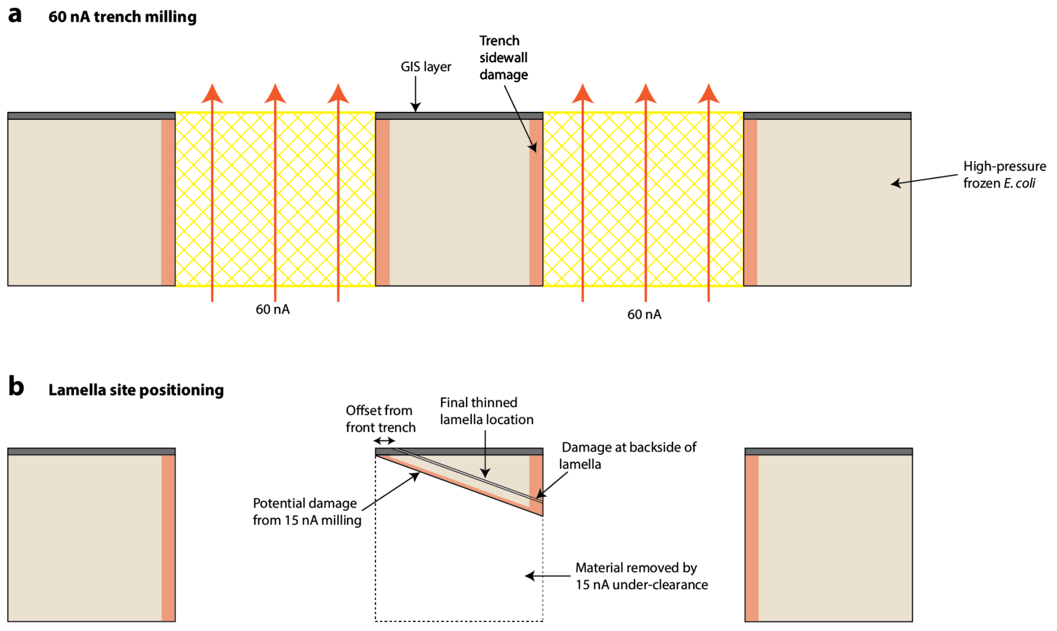


**Supplementary Figure 8. Proposed model for amorphous and striated layers on backside of FIB lamella. a** Trenches are milled with 60 nA xenon from the backside of the grid (ablated material indicated in yellow, direction of milling indicated by orange arrows). We speculate this may cause a region of amorphisation to the side walls of the trenches (red). **b** Following clearance of material under the lamella site at 15 nA, the final lamella site is positioned, with the front edge typically slightly offset from the front trench. The shallow milling angle therefore means that the final lamella will include any damaged sample material (red) on the back side, but not the front.

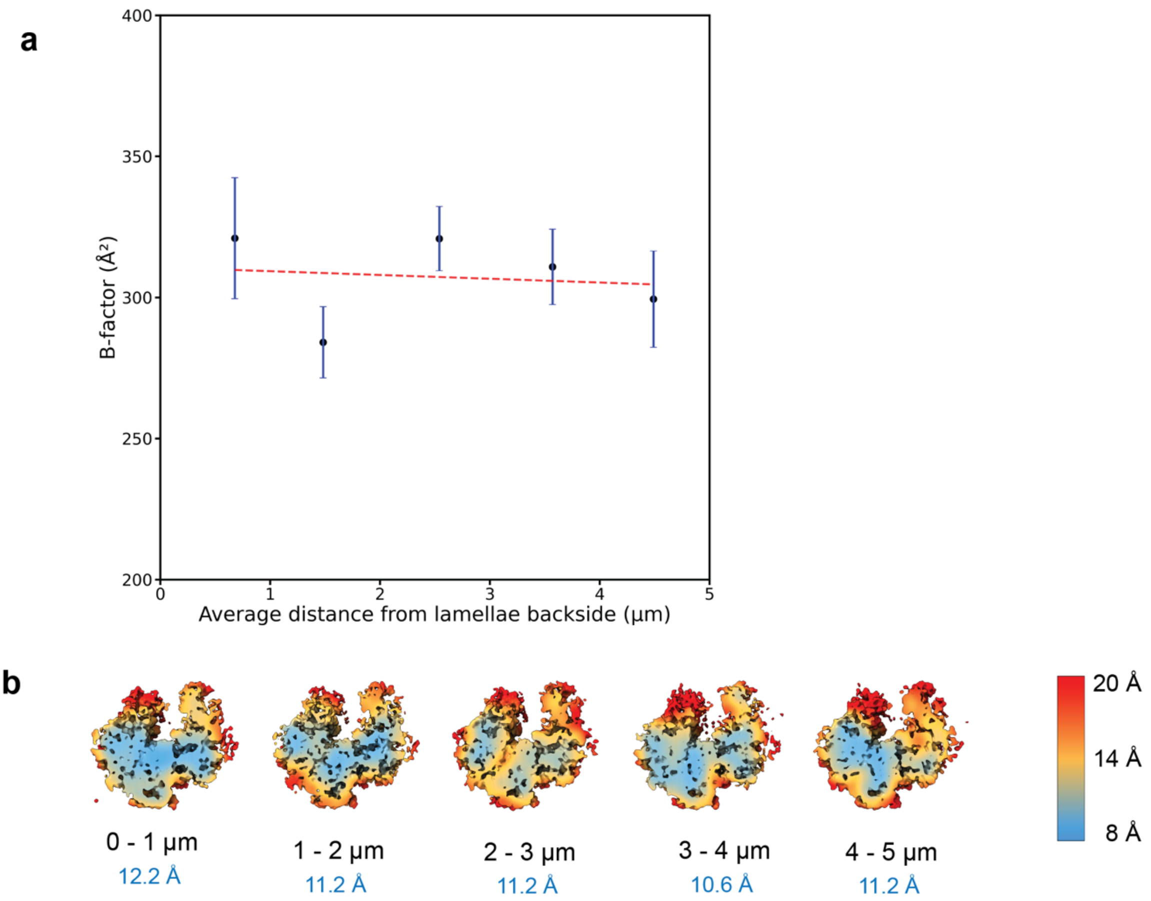


**Supplementary Figure 9. Effect of distance to backside of lamellae on ribosome STA. a** Average distance (μm) from backside of lamellae for particles within 5 μm of backside vs B-factor value (mean ± SE) per group. No significant correlation was observed (Spearman correlation: r = -0.40 p-value = 0.50). n=3 technical replicates **b** Local resolution maps for backside damage distance groups, for 800 particles. One of n=3 repeats depicted. Global resolution estimates listed in blue. Source data are provided as a Source Data File.

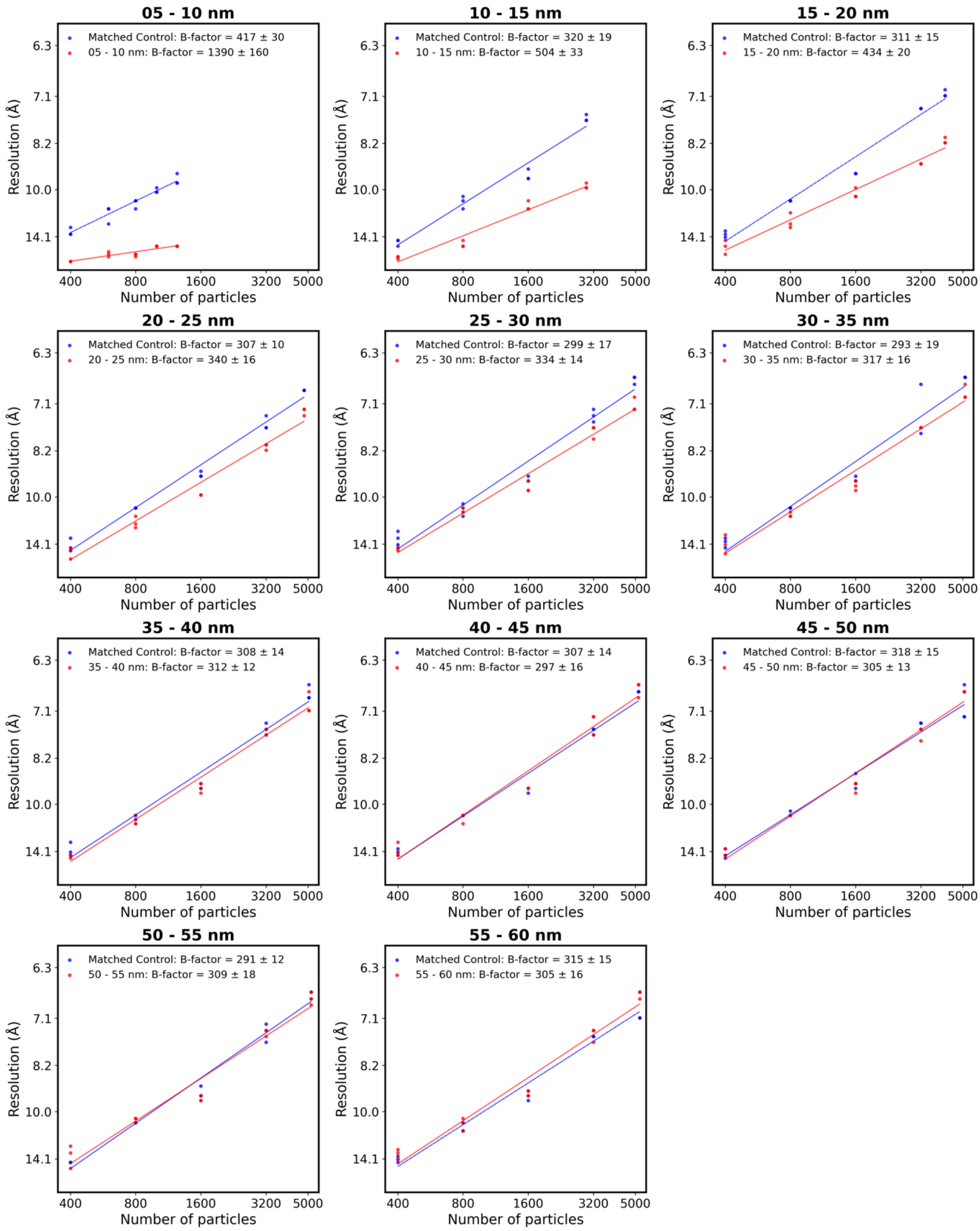


**Supplementary Figure 10.** B-factor plots of FIB surface damage groups. B-factor plots for ribosome particles grouped by distance from the PFIB milling surfaces (blue) and their corresponding matched controls of ribosome particles located further from the milling surfaces (orange). All resolution measurements from n=3 B-factor jobs are plotted. B-factors reported as mean ± SE, calculated as described in Methods. Source data are provided as a Source Data File.
