# Supplementary material for: Xenon plasma focused ion beam lamella fabrication on high-pressure frozen specimens for structural cell biology
Source: Nat Commun. 2025 Mar 7;16:2286. doi: 10.1038/s41467-025-57493-3 (PMC11889171; doi:10.1038/s41467-025-57493-3)
Supplement: Supplementary file 2 — Description of Additional Supplementary Files [file 41467_2025_57493_MOESM2_ESM.pdf]

Description of Additional Supplementary Files

File Name: Supplementary Movie 1

Description: Removal of Ice contamination from the grid, by imaging with the FIB beam at low magnification.

File Name: Supplementary Movie 2

Description: Tomograms recorded on lamellae with variable local thicknesses, for which a single slice is shown in Figure 2c-e. Scalebar: 100 nm.

File Name: Supplementary Movie 3

Description: Movie of consensus STA density map of E. coli 70S ribosome surfaces coloured by local resolution. Scalebar: 1 nm. Colour key used is the same as that used in Figure 2.

File Name: Supplementary Movie 4

Description: Aligned tilt-series of the striated damage pattern at the back of a lamella shown in Figure 3b. Scalebar: 100 nm.

File Name: Supplementary Movie 5

Description: Reconstructed tomogram of the striated damage pattern at the back of a lamella, from the tilt-series shown in Figure 3b and Supplementary Video 3. Considerable reconstruction artefacts are present at the very back of the lamella, visible as streaks extending from the edges of high-contrast bands of the damaged area. Scalebar: 100 nm.
