# Supplementary material for: Xenon plasma focused ion beam lamella fabrication on high-pressure frozen specimens for structural cell biology
Source: Nat Commun. 2025 Mar 7;16:2286. doi: 10.1038/s41467-025-57493-3 (PMC11889171; doi:10.1038/s41467-025-57493-3)
Supplement: Supplementary file 8 — Reporting Summary [file 41467_2025_57493_MOESM8_ESM.pdf]

nature portfolio

Corresponding author(s): Michael Grange

Last updated by author(s): Jan 13, 2025

Reporting Summary

Nature Portfolio wishes to improve the reproducibility of the work that we publish. This form provides structure for consistency and transparency in reporting. For further information on Nature Portfolio policies, see our Editorial Policies and the Editorial Policy Checklist.

Statistics

For all statistical analyses, confirm that the following items are present in the figure legend, table legend, main text, or Methods section.

n/a	Confirmed	
☐	☒	The exact sample size (*n*) for each experimental group/condition, given as a discrete number and unit of measurement
☐	☒	A statement on whether measurements were taken from distinct samples or whether the same sample was measured repeatedly
☐	☒	The statistical test(s) used AND whether they are one- or two-sided<br>*Only common tests should be described solely by name; describe more complex techniques in the Methods section.*
☒	☐	A description of all covariates tested
☒	☐	A description of any assumptions or corrections, such as tests of normality and adjustment for multiple comparisons
☐	☒	A full description of the statistical parameters including central tendency (e.g. means) or other basic estimates (e.g. regression coefficient) AND variation (e.g. standard deviation) or associated estimates of uncertainty (e.g. confidence intervals)
☐	☒	For null hypothesis testing, the test statistic (e.g. *F*, *t*, *r*) with confidence intervals, effect sizes, degrees of freedom and *P* value noted<br>*Give P values as exact values whenever suitable.*
☒	☐	For Bayesian analysis, information on the choice of priors and Markov chain Monte Carlo settings
☒	☐	For hierarchical and complex designs, identification of the appropriate level for tests and full reporting of outcomes
☐	☒	Estimates of effect sizes (e.g. Cohen's *d*, Pearson's *r*), indicating how they were calculated

Our web collection on statistics for biologists contains articles on many of the points above.

Software and code

Policy information about availability of computer code

Data collection	Tomo 5 Software (Thermo Fisher Scientific)<br>Auto TEM cryo 2.4 (Thermo FIsher Scientific)
Data analysis	Warp/M (http://warpem.com/warp/#)<br>crYOLO (https://cryolo.readthedocs.io/en/latest/)<br>RELION (https://relion.readthedocs.io/en/release-4.0/)<br>IMOD 4.11.1(https://bio3d.colorado.edu/imod/)<br>EMAN2 (https://blake.bcm.edu/emanwiki/EMAN2/Install/BinaryInstallAnaconda/2.91)<br>https://github.com/rosalindfranklininstitute/RiboDist<br>Fiji is just imageJ (https://imagej.net/software/fiji/downloads)<br>ISONet (https://github.com/IsoNet-cryoET/IsoNet)<br>AreTomo 1.2.5 (https://msg.ucsf.edu/software)<br>ChimeraX (https://www.rbvi.ucsf.edu/chimerax/)<br>StarParser (https://github.com/sami-chaaban/starparser)<br>Python (https://www.python.org)<br>R (https://www.r-project.org)

For manuscripts utilizing custom algorithms or software that are central to the research but not yet described in published literature, software must be made available to editors and reviewers. We strongly encourage code deposition in a community repository (e.g. GitHub). See the Nature Portfolio guidelines for submitting code & software for further information.

Data

Policy information about availability of data

All manuscripts must include a data availability statement. This statement should provide the following information, where applicable:

- Accession codes, unique identifiers, or web links for publicly available datasets
- A description of any restrictions on data availability
- For clinical datasets or third party data, please ensure that the statement adheres to our policy

Subtomogram averages of ribosomes obtained in this study were deposited in the Electron Microscopy Data Bank (EMDB): full reconstruction (EMD-50675), the reconstructions from the 1 µm distance groups from the backside damage (EMD-50699, EMD-50700, EMD-50701, EMD-50702, EMD-50703), 5-nm distance groups for the PFIB surface damage (EMD-50676, EMD-50678, EMD-50679, EMD-50680, EMD-50681, EMD-50682, EMD-50683, EMD-50684, EMD-50685, EMD-50686, EMD-50687) and the matched controls (EMD-50688, EMD-50689, EMD-50690, EMD-50691, EMD-50692, EMD-50693, EMD-50694, EMD-50695, EMD-50696, EMD-50697, EMD-50698), and the 10,000 particle sets for determination of ion beam damage impact (EMD-52177, EMD-52178, EMD-52179). The raw microscope data (frames and metadata files) used for subtomogram averaging and associated processed data are available on the EMPIAR data server under accession code EMPIAR-12413. Source data for the indicated panels are provided with this paper.

Research involving human participants, their data, or biological material

Policy information about studies with human participants or human data. See also policy information about sex, gender (identity/presentation), and sexual orientation and race, ethnicity and racism.

Reporting on sex and gender	N/A
Reporting on race, ethnicity, or other socially relevant groupings	N/A
Population characteristics	N/A
Recruitment	N/A
Ethics oversight	N/<br>N/A

Note that full information on the approval of the study protocol must also be provided in the manuscript.

Field-specific reporting

Please select the one below that is the best fit for your research. If you are not sure, read the appropriate sections before making your selection.

☒ Life sciences ☐ Behavioural & social sciences ☐ Ecological, evolutionary & environmental sciences

For a reference copy of the document with all sections, see nature.com/documents/nr-reporting-summary-flat.pdf

Life sciences study design

All studies must disclose on these points even when the disclosure is negative.

Sample size	No sample size calculations were performed.
Data exclusions	106 out of 286 tomograms were excluded from further processing using the following criteria: 1) the contrast of the tomogram did not allow for a confident annotation of the milling surfaces, 2) ribosomes could not be clearly identified in the tomogram, or 3) the tomogram contained substantial redeposition or other surface contamination. Particles positions were imported into M and an initial round of M was run with no refinement parameters selected to verify that the programme could run successfully. An additional 13 tomograms were excluded during M processing due to software errors.
Replication	Experimental repeats were performed where appropriate
Randomization	N/A
Blinding	No blinding was performed

Reporting for specific materials, systems and methods

We require information from authors about some types of materials, experimental systems and methods used in many studies. Here, indicate whether each material, system or method listed is relevant to your study. If you are not sure if a list item applies to your research, read the appropriate section before selecting a response.

Materials & experimental systems

n/a	Involved in the study
☒	☐ Antibodies
☐	☒ Eukaryotic cell lines
☒	☐ Palaeontology and archaeology
☒	☐ Animals and other organisms
☒	☐ Clinical data
☒	☐ Dual use research of concern
☒	☐ Plants

Methods

n/a	Involved in the study
☒	☐ ChIP-seq
☒	☐ Flow cytometry
☒	☐ MRI-based neuroimaging

Eukaryotic cell lines

Policy information about cell lines and Sex and Gender in Research

Cell line source(s)	Yeast
Authentication	*Describe the authentication procedures for each cell line used OR declare that none of the cell lines used were authenticated.*
Mycoplasma contamination	*Confirm that all cell lines tested negative for mycoplasma contamination OR describe the results of the testing for mycoplasma contamination OR declare that the cell lines were not tested for mycoplasma contamination.*
Commonly misidentified lines (See ICLAC register)	*Name any commonly misidentified cell lines used in the study and provide a rationale for their use.*

Plants

Seed stocks	*Report on the source of all seed stocks or other plant material used. If applicable, state the seed stock centre and catalogue number. If plant specimens were collected from the field, describe the collection location, date and sampling procedures.*
Novel plant genotypes	*Describe the methods by which all novel plant genotypes were produced. This includes those generated by transgenic approaches, gene editing, chemical/radiation-based mutagenesis and hybridization. For transgenic lines, describe the transformation method, the number of independent lines analyzed and the generation upon which experiments were performed. For gene-edited lines, describe the editor used, the endogenous sequence targeted for editing, the targeting guide RNA sequence (if applicable) and how the editor was applied.*
Authentication	*Describe any authentication procedures for each seed stock used or novel genotype generated. Describe any experiments used to assess the effect of a mutation and, where applicable, how potential secondary effects (e.g. second site T-DNA insertions, mosiacism, off-target gene editing) were examined.*
